# A key role for tetrahydrobiopterin‐dependent endothelial NOS regulation in resistance arteries: studies in endothelial cell tetrahydrobiopterin‐deficient mice

**DOI:** 10.1111/bph.13728

**Published:** 2017-03-13

**Authors:** Surawee Chuaiphichai, Mark J Crabtree, Eileen Mcneill, Ashley B Hale, Lucy Trelfa, Keith M Channon, Gillian Douglas

**Affiliations:** ^1^ British Heart Foundation Centre of Research Excellence, Division of Cardiovascular MedicineUniversity of OxfordOxfordUK; ^2^ Wellcome Trust Centre for Human GeneticsUniversity of OxfordOxfordUK

## Abstract

**Background and Purpose:**

The cofactor tetrahydrobiopterin (BH4) is a critical regulator of endothelial NOS (eNOS) function, eNOS‐derived NO and ROS signalling in vascular physiology. To determine the physiological requirement for *de novo* endothelial cell BH4 synthesis for the vasomotor function of resistance arteries, we have generated a mouse model with endothelial cell‐specific deletion of *Gch1,* encoding GTP cyclohydrolase 1 (GTPCH), an essential enzyme for BH4 biosynthesis, and evaluated BH4‐dependent eNOS regulation, eNOS‐derived NO and ROS generation.

**Experimental Approach:**

The reactivity of mouse second‐order mesenteric arteries was assessed by wire myography. High performance liquid chromatography was used to determine BH4, BH2 and biopterin. Western blotting was used for expression analysis.

**Key Results:**

*Gch1* ^*fl/fl*^Tie2cre mice demonstrated reduced GTPCH protein and BH4 levels in mesenteric arteries. Deficiency in endothelial cell BH4 leads to eNOS uncoupling, increased ROS production and loss of NO generation in mesenteric arteries of *Gch1* ^*fl/fl*^Tie2cre mice. *Gch1* ^*fl/fl*^Tie2cre mesenteric arteries had enhanced vasoconstriction to U46619 and phenylephrine, which was abolished by L‐NAME. Endothelium‐dependent vasodilatations to ACh and SLIGRL were impaired in mesenteric arteries from *Gch1* ^*fl/fl*^Tie2cre mice, compared with those from wild‐type littermates. Loss of eNOS‐derived NO‐mediated vasodilatation was associated with increased eNOS‐derived H_2_O_2_ and cyclooxygenase‐derived vasodilator in *Gch1* ^*fl/fl*^Tie2cre mesenteric arteries.

**Conclusions and Implications:**

Endothelial cell *Gch1* and BH4‐dependent eNOS regulation play pivotal roles in maintaining vascular homeostasis in resistance arteries. Therefore, targeting vascular *Gch1* and BH4 biosynthesis may provide a novel therapeutic target for the prevention and treatment of microvascular dysfunction in patients with cardiovascular disease.

**Abbreviations**
BH4: tetrahydrobiopterin
DHE: dihydroethidium
EDHF: endothelium‐derived hyperpolarizing factor
eNOS: endothelial NOS
GTPCH: GTP cyclohydrolase 1
PEG‐catalase: catalase‐polyethylene glycol conjugate
SNP: sodium nitroprusside

## Tables of Links

**Table:** 

TARGETS	
**Enzymes** ^*a*^	**GPCRs** ^*c*^
Cyclooxygenase	α_1_‐adrenoceptor
eNOS	Proteinase‐activated receptor 2 (PAR2)
**Voltage‐gated ion channels** ^*b*^	TxA_2_ receptor
K_Ca_2.3 (SK_Ca_)	
K_Ca_3.1 (IK_Ca_)	

LIGANDS	
ACh	Nitric oxide (NO)
Apamin	Phenylephrine
Charybdotoxin	SLIGRL
H_2_O_2_	Tetrahydrobiopterin
Indomethacin	U46619
L‐NAME	

These Tables list key protein targets and ligands in this article that are hyperlinked to corresponding entries in http://www.guidetopharmacology.org, the common portal for data from the IUPHAR/BPS Guide to PHARMACOLOGY (Southan *et al*., 2016), and are permanently archived in the Concise Guide to PHARMACOLOGY 2015/16 (^*a,b,c*^Alexander *et al*., 2015a,b,c).

## Introduction

Hypertension affects more than one in four adults in the UK and is a major risk factor for heart failure, kidney disease and stroke. Hypertension is associated with endothelial dysfunction and impaired endothelium‐dependent relaxation. In a normal vasculature, endothelium‐dependent relaxation is mediated by three principal vasodilators: NO, (Furchgott and Zawadzki, 1980; Palmer *et al.*, 1988), prostacyclin (Moncada *et al*., 1976) and endothelium‐derived hyperpolarizing factor (EDHF) (Busse *et al*., 2002). The relative contribution of NO, prostacyclin and EDHF to the dilator response is not fixed and compensatory changes have been observed in several disease states (Matoba *et al*., 2000; Scotland *et al*., 2005; Takaki *et al*., 2008).

Tetrahydrobiopterin (BH4) is a critical regulator of endothelial NOS (eNOS) function, and eNOS‐derived NO and ROS signalling in vascular physiology. Biosynthesis of BH4 is catalysed by GTP cyclohydrolase 1 (GTPCH), a rate limiting enzyme for *de novo* BH4 biosynthesis, which is encoded by *Gch1*. We have previously shown that *Gch1* expression is a key determinant of BH4 bioavailability, eNOS regulation and thus of NO generation (Vasquez‐Vivar *et al*., 2002; Crabtree *et al*., 2009a). When vascular BH4 bioavailability is limited, eNOS is unable to generate NO from L‐arginine and becomes ‘uncoupled’, resulting in generation of superoxide anion and other ROS, rather than NO. Recent evidence indicates that decreased bioavailability of vascular BH4 contributes to the pathogenesis of endothelial dysfunction and vascular disease states, such as hyperlipidaemia (Mollnau *et al*., 2003), diabetes (Hink *et al*., 2001) and hypertension (Landmesser *et al*., 2003; Li *et al*., 2006; Chuaiphichai *et al*., 2014). Clinically, a common *GCH1* variant, C + 243 T in the 3′‐ untranslated region, has been associated with decreased NO production and increased blood pressure (Zhang *et al*., 2007). Studies of a haplotype defined by three single nucleotide polymorphisms in *GCH1* have shown that the X haplotype is associated with lower vascular levels of *GCH1* mRNA and decreased levels of BH4 in the vasculature. This attenuation of BH4 was associated with increased vascular superoxide production and reduced endothelial‐dependent vasodilatation in arterial and venous segments from patients with coronary artery disease (Antoniades *et al*., 2008). Taken together, these studies imply a key role for *GCH1* in vascular homeostasis.

The results from these clinical studies have been supported by findings in preclinical models. Treatment of mice with a selective inhibitor of GTPCH, 2,4, diamino‐6‐hydroxypyrimidine, resulted in endothelial dysfunction and ultimately hypertension (Mitchell *et al*., 2003). In addition, mice with siRNA knockdown of *Gch1* exhibit decreased vascular BH4 levels, eNOS uncoupling and increased blood pressure (Wang *et al*., 2008). Recently, we have reported that endothelial cell‐targeted *Gch1* knockout (*Gch1* ^*fl/fl*^Tie2cre mouse) results in endothelial cell‐specific BH4 deficiency and hypertension (Chuaiphichai *et al*., 2014). However, the mechanism by which an endothelial cell‐specific deficiency in *Gch1* alters resistance artery function remains unknown. In addition, the effect of this BH4‐dependent alteration in NO/ROS generation on the development of vascular dysfunction in resistance arteries has also yet to be elucidated. This is important as resistance arteries are the major site for blood pressure regulation. To address these questions, we have utilized a mouse model of endothelial cell‐specific deletion of BH4.

## Methods

### Animals

All animal care and experimental studies were in accordance with the UK Home Office regulations (Guidance on the Operation of Animals, Scientific Procedures Act, 1986) and were approved by the Local Ethical Review Committee. Animal studies are reported in compliance with the ARRIVE guidelines (Kilkenny *et al.*, 2010; McGrath and Lilley, 2015). Mice were housed in a specific pathogen‐free environment in Tecniplast Sealsafe IVC cages (floor area 542cm^2^) with a maximum of six other mice. Woodchip and Sizzle‐Nest bedding and environmental enrichment, for example, houses and tunnels were included in every cage. Mice were kept in a 12 h light/dark cycle and in controlled temperatures (20–22°C) and fed normal chow and water *ad libitum*.

### Gch1 knockout mice

We have generated a *Gch1* conditional knockout (floxed) allele using Cre/loxP strategy as described previously (Chuaiphichai *et al*., 2014). *Gch1* ^*fl/fl*^ animals were bred with Tie2cre transgenic mice to produce *Gch1* ^*fl/fl*^Tie2cre mice where *Gch1* is deleted in endothelial cells, generating a mouse model of endothelial cell‐specific BH4 deficiency. *Gch1* ^*fl/fl*^Tie2cre mice are to date the only model available to study the effect of endothelial cell BH4 deficiency. The Tie2cre transgene is active in the female germline. Consequently only male animals are used to establish breeding pairs to maintain conditional expression. Mice were genotyped according to the published protocol (Chuaiphichai *et al*., 2014; Chuaiphichai *et al*., 2016). Adult male *Gch1* ^*fl/fl*^Tie2cre and their *Gch1* ^*fl/fl*^ (wild‐type) littermates on a pure (>10 generations) C57BL6/J background were bred in house and were used for all experiments at 12 to 22 weeks.

### Determination of tissue BH4 levels

BH4 and oxidized biopterins (BH2 and biopterin) were determined by HPLC followed by electrochemical and fluorescence detection, respectively, following an established protocol (Crabtree *et al*., 2009b). Briefly, mesenteric arteries were freeze‐thawed in ice‐cold resuspension buffer (50 mmol·L^−1^ phosphate‐buffered saline, 1 mmol·L^−1^ dithioerythriol, 1 mmol·L^−1^ EDTA, pH 7.4). After centrifugation at 17,000 x g for 10 min at 4°C, supernatant was removed, and ice‐cold acid precipitation buffer (1 mol·L^−1^ phosphoric acid, 2 mol·L^−1^ trichloroacetic acid, 1 mmol·L^−1^ dithioerythritol) was added. Following centrifugation at 17,000 x g for 10 min at 4°C, the supernatant was removed and injected into the HPLC system. Quantification of BH4 and oxidized biopterins was obtained by comparison with external standards and normalized to protein concentration, determined by the bicinchoninic acid protein assay.

### Isometric tension vasomotor studies

Vasomotor function was analysed using isometric tension studies in a wire myograph (Multi‐Myograph 610 M, Danish Myo Technology, Denmark). Briefly, mice were killed by overdose of inhaled isoflurane. The mesentery was excised from the mouse and placed in cool Krebs–Henseleit buffer [KHB (in mmol·L^−1^): NaCl 120, KCl 4.7, MgSO_4_ 1.2, KH_2_PO_4_ 1.2, CaCl_2_ 2.5, NaHCO_3_ 25, glucose 5.5]. Segments of second‐order artery were carefully dissected free from surrounding fat and connective tissue, as described previously(Chuaiphichai *et al*., 2014; Chuaiphichai *et al*., 2016). The mesenteric arteries (2 mm) were mounted on a wire myograph containing 5 mL of KHB at 37°C, gassed with 95% O_2_/5% CO_2_. After allowing vessels to equilibrate for 30 min, the passive tension‐internal circumference was determined by stretching to achieve an internal circumference equivalent to 90% of that of the blood vessel under transmural pressure of 100 mmHg. The vessel viability was tested using 45 mmol·L^−1^ KCl. Concentration‐response contraction curves were established using cumulative half‐log concentrations of phenylephrine and U46619 in the presence or absence of 100 μmol·L^−1^ of non‐selective NOS inhibitor, L‐NAME. Vessels were washed three times with fresh KHB, equilibrated for 20 min and then precontracted to approximately 80% of maximal tension with U46619. ACh (1 nmol·L^−1^–10 μmol·L^−1^) and SLIGRL (10 nmol·L^−1^–10 μmol·L^−1^) were used to stimulate endothelium‐dependent vasodilatations in increasing cumulative concentrations. Responses were expressed as a percentage of the precontracted tension. Finally, the NO donor sodium nitroprusside (SNP, 0.1 nmol·L^−1^–1 μmol·L^−1^) was used to test endothelium‐independent smooth muscle relaxation in the presence of L‐NAME. In addition, endothelium‐dependent vasodilatation to ACh and SLIRGL was examined after 20 min incubation with different combinations of indomethacin (10 μmol·L^−1^), a non‐selective cyclooxygenase inhibitor, L‐NAME (100 μmol·L^−1^), apamin (50 nmol·L^−1^), to block small‐conductance Ca^2+^‐activated K^+^ channels, charybdotoxin (100 nmol·L^−1^), to block, non‐selectively, intermediate and large‐conductance Ca^2+^‐activated K^+^ channels, catalase‐polyethylene glycol (PEG‐catalase; 400 units·mL^−1^), a cell permeable H_2_O_2_ scavenger, SOD‐PEG (200 units·mL^−1^), a cell permeable superoxide scavenger and sepiapterin (100 μmol·L^−1^), a BH4 precursor.

### Oxidative fluorescent microtopography

Endothelial ROS production in mouse second‐order mesenteric frozen sections (*n* = 6 to 7 animals per group) was detected using dihydroethidium (DHE) and fluorescent microscopy, as previously described (Bendall *et al*., 2005). Briefly, second‐order mesenteric arteries (*n* = 6 per group) were harvested and frozen in optimal cutting temperature compound (VWR International Ltd). Cryosections (14 μm) were cut at −25°C using a cryostat and placed on poly‐lysine coated glass slide. Sections were incubated in KHB with or without 200 μmol·L^−1^ L‐NAME for 20 min at 37°C, then with 2 μM DHE (Invitrogen) for 20 min at 37°C in darkness. Sections were washed with ice‐cold KHB, cover‐slipped and placed in darkness. Images (×40) were acquired using a laser confocal microscope, set at identical acquisition settings (488 nm Excitation and 585 nm Emission). Endothelial ROS production was quantified using Image‐Pro Plus software by measuring the sum of the red intensities of the cells on the luminal side of the internal elastic lamina divided by the length of the vessel.

### Nitrite and nitrate determination

The levels of nitrite/nitrate (NO_x_) produced by mesenteric arteries were determined using the CLD88 NO analyser (Ecophysics), as previously described (Chuaiphichai *et al*., 2016). Briefly, freshly isolated mesenteric arteries were pooled and stimulated with 1 μmol·L^−1^ ACh for 24 h at 37°C. To measure NO_x_ accumulation, conditioned medium was collected and assayed. Quantification of NO_x_ accumulation was obtained by comparison with external standards and normalized to protein concentration, determined by the bicinchoninic acid protein assay.

### Cell culture

*Gch Knockdown by RNA Interference*—*Gch*‐specific, ON‐TARGETplus SMARTpool siRNA was purchased from Dharmacon Thermo Scientific. sEnd.1 endothelial cells (kindly donated by Dr. Patrick Vallance, University College, London) were grown in Dulbecco's modified Eagle's medium (Invitrogen) supplemented with glutamine (2 mmol·L^−1^) penicillin (100 units mL) and streptomycin (0.1 mg mL). About 24 h prior to transfection, the cells were seeded into 6‐well plates. The cells were then transfected with *Gch*‐specific siRNA (100 nmol·L^−1^), nonspecific pooled duplex negative control siRNA (100 nmol·L^−1^). The cells were cultured for 72 h, and gene silencing was detected by analysis of GTPCH protein expression by Western blotting using GTPCH‐specific antibodies.

### Western blotting

Western blots were performed to evaluate protein levels of GTPCH, eNOS and antioxidant proteins in mesenteric tissues from wild‐type and *Gch1* ^*fl/fl*^Tie2cre mice. The proteins extracted (15 μg) from mesenteric tissue were loaded into a NuPage 4–12% Bis‐Tris gel, transferred to PVDF membrane and incubated with a 1:10 000 dilution of rabbit anti‐mouse GTPCH antibody (a gift from Prof Steve Gross). Other proteins were detected using a 1:5000 dilution of mouse anti‐eNOS antibody (BD Transduction Laboratories), 1:500 dilution of rabbit anti‐phospho Ser^1177^‐eNOS antibody (Cell Signalling), 1:500 dilution of rabbit anti‐phospho Thr^495^‐eNOS antibody (Cell Signalling), 1:500 dilution of goat anti‐mouse CD102 antibody (R&D Systems), 1:2500 dilution of rabbit anti‐catalase antibody (Calbiochem), 1:2500 dilution of rabbit anti‐MnSOD antibody (Stressgen Bioreaents), 1:2500 dilution of rabbit anti‐ecSOD antibody (Stressgen Bioreaents), 1:2500 dilution of rabbit anti‐Cu/ZnSOD antibody (Stressgen Bioreaents) and 1:20 000 dilution of rabbit anti‐β tubulin antibody (Abcam) followed by appropriate HRP‐conjugated secondary antibody (Promega). Protein bands were visualized by enhanced chemiluminescence (Super West Pico Chemiluminescence, Thermo Scientific).

### Monomer and dimer

Low‐temperature SDS‐PAGE was performed for detection of the eNOS monomer and dimer as described previously (Cai *et al*., 2002; Benson *et al*., 2013).

### Immunodetection of eNOS glutathionylation

eNOS glutathionylation was examined in cell lysates by immunoprecipitation as described previously (Crabtree *et al*., 2013).

### Data and statistical analysis

The data and statistical analysis comply with the recommendations on experimental design and analysis in pharmacology (Curtis *et al.*, 2015). Data are expressed as means ± SEM and analysed using Graphpad Prism version 5.0 (San Diego, USA). Comparisons between WT and *Gch1* ^*fl/fl*^Tie2cre were made by unpaired *Student's t*‐test. Concentration‐response curves were compared by two‐way ANOVA for repeated measurements. A *P*‐value of less than 0.05 was considered statistically significant. After genotyping, mice were randomly assigned to experiments. Both the operator and data analyst were blinded to genotype. All mice were re‐genotyped at the end of the experiment.

### Materials

All drugs were obtained from Sigma‐Aldrich (Poole, UK) with the exception of sepiapterin (Schircks Laboratories, Switzerland), SLIGRL (Abcam, UK). All drugs were dissolved in distilled water, with the exception of indomethacin, which was dissolved in ethanol and U46619 and sepiapterin, which were dissolved in DMSO.

## Results

### Endothelial cell‐targeted *Gch1* deletion leads to BH4 deficiency in endothelial cells of mesenteric arteries

**Figure 1:**
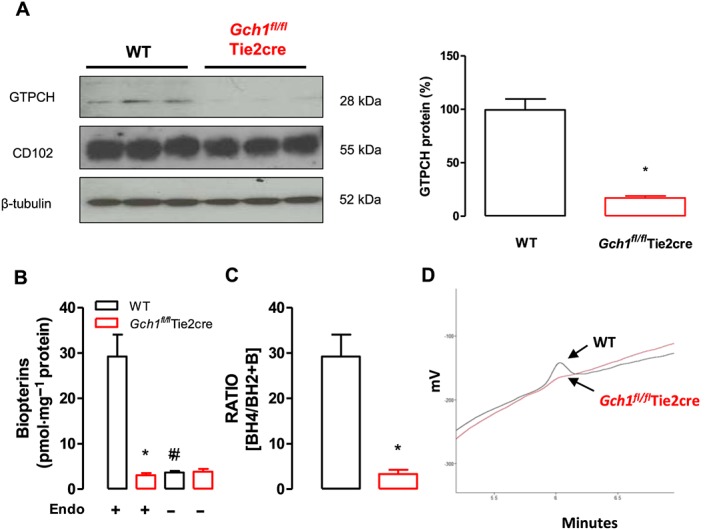
GTPCH and eNOS protein expression in mesenteric arteries. (A) Representative immunoblots showing GTPCH protein in wild‐type (WT) and *Gch1* ^*fl/fl*^Tie2cre mesenteric arteries, with quantitative data, measured as percentage band density, right and corresponding CD102 (endothelial cell marker) and β‐tubulin immunoblots, below. *P < 0.05; significantly different from WT; *n* = 6 animals per group. (B) Vascular BH4, measured by HPLC, was significantly reduced in mesenteric arteries from *Gch1* ^*fl/fl*^Tie2cre mice compared with WT littermates. Removal of endothelium in mesenteric arteries significantly reduced vascular BH4 levels in WT mice but unchanged in *Gch1* ^*fl/fl*^Tie2cre mice. *P < 0.05; significantly different from WT; #*P <* 0.05, significantly different from Endo+ ; *n* = 5 WT and six *Gch1* ^*fl/fl*^Tie2cre mice, only five WT matched littermates were available) (C) Ratio of BH4 to BH2 was significantly reduced in mesenteric arteries from *Gch1* ^*fl/fl*^Tie2cre mice compared with WT littermate controls. *P < 0.05; significantly different from WT; *n* = 5 WT and six *Gch1* ^*fl/fl*^Tie2cre mice, only five WT matched littermates were available). (D) Representative chromatograms of BH4 traces in mesenteric arteries from WT and *Gch1* ^*fl/fl*^Tie2cre mice.

Western blot analysis showed that GTPCH protein was barely detectable in mesenteric arteries from *Gch1* ^*fl/fl*^Tie2cre mice, whereas this protein was readily detected in wild‐type mesenteric arteries (Figure 1A). The endothelial cell‐surface marker CD102 and loading control β‐tubulin were present equally in mesenteric arteries from both wild‐type and *Gch1* ^*fl/fl*^Tie2cre mice (Figure 1A). In *Gch1* ^*fl/fl*^Tie2cre mice, vascular BH4 levels and the ratio of BH4 to 7,8‐BH2, as determined by HPLC, were significantly decreased in mesenteric arteries compared with wild‐type littermate controls (Figure 1B–D). In endothelial‐denuded mesenteric arteries, vascular BH4 levels were decreased by ≈90% in mesenteric arteries from wild‐type mice such that vascular BH4 levels were no longer different between wild‐type and *Gch1* ^*fl/fl*^Tie2cre mice, suggesting the endothelium is the principal site for BH4 biosynthesis in mesenteric arteries (Figure 1B). Importantly, removal of the endothelium had no significant effect on vascular BH4 levels in mesenteric arteries from *Gch1* ^*fl/fl*^Tie2cre mice (Figure 1B), indicating a complete deletion of the endothelial cell *Gch1* gene and subsequently decreased GTPCH protein and BH4 levels in mesenteric arteries with Tie2. This finding demonstrates that endothelial cell‐targeted *Gch1* deletion leads to endothelial cell BH4 deficiency in mesenteric arteries of *Gch1* ^*fl/fl*^Tie2cre mice.

### Endothelial cell BH4 deficiency leads to eNOS uncoupling with decreased NO generation and increased ROS production in mesenteric arteries

**Figure 2:**
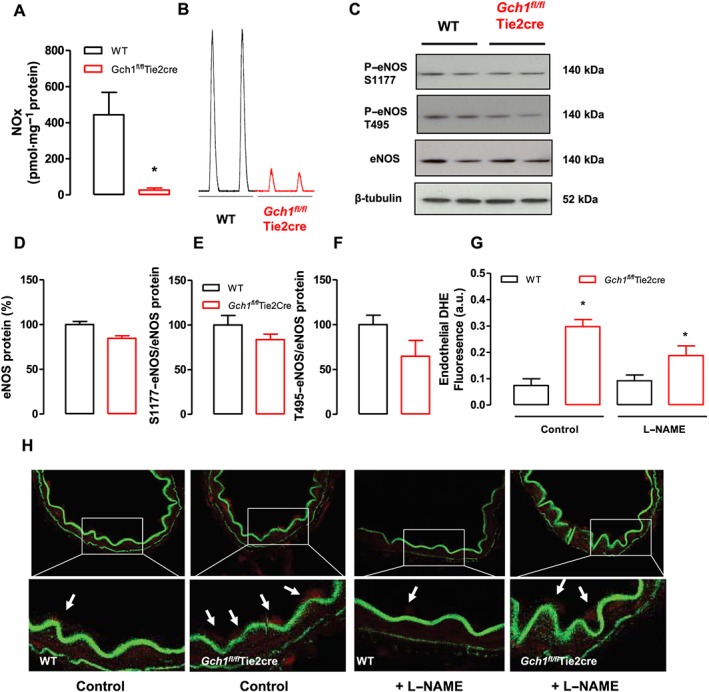
NO bioactivity and ROS generation in mesenteric arteries from *Gch1* ^*fl/fl*^Tie2cre and WT littermates. (A) NO_x_ accumulation in isolated mesenteric arteries stimulated with 1 μmol·L^−1^ ACh for 24 h. NO_x_ accumulation in stimulated mesenteric arteries from *Gch1* ^*fl/fl*^Tie2cre mice was decreased compared with that from WT controls. *P < 0.05; significantly different from WT; *n* = 5 WT and six *Gch1* ^*fl/fl*^Tie2cre mice, only five WT matched littermates were available). (B) Representative traces of NO_x_ accumulation in mesenteric arteries from WT and *Gch1* ^*fl/fl*^Tie2cre mice. (C) Representative immunoblots showing phosphorylation of eNOS at Ser^1177^, Thr^495^, eNOS and β‐tubulin (as loading control) in isolated second‐order mesenteric arteries from WT and *Gch1* ^*fl/fl*^Tie2cre mice. (D, E and F) Quantification data, measured as band density of eNOS protein, S1177‐eNOS to total eNOS protein and T495‐eNOS to total eNOS protein respectively (*n* = 6 animals per group). (G) DHE fluorescence to measure *in situ* endothelial ROS production in frozen sections of second‐order mesenteric arteries from *Gch1* ^*fl/fl*^Tie2cre and WT mice. *P < 0.05, significantly different from WT; *n* = 5 WT and six *Gch1* ^*fl/fl*^Tie2cre, one mesenteric artery from a WT mouse could not be sectioned) (H) Representative mesenteric sections (×40) showing red endothelial cells (arrows). Endothelium‐derived ROS production, quantified in arbitrary units as area of luminal red staining/length of luminal surface, in the absence and presence of 200 μmol·L^−1^ L‐NAME.

To examine the effects of endothelial cell‐specific BH4 deficiency on NO bioactivity in isolated mesenteric arteries, the levels of NO_x_ produced by mesenteric arteries stimulated with ACh (1 μmol·L^−1^, 24 h) were determined by the NO analyser. The levels of NO_x_ accumulation produced by mesenteric arteries from *Gch1* ^*fl/fl*^Tie2cre mice were reduced compared with wild‐type mice (Figure 2A, B). Despite the reduction in NO bioactivity, eNOS protein levels assessed by Western blot were comparable between wild‐type and *Gch1* ^*fl/fl*^Tie2cre mesenteric arteries (Figure 2C, D). Furthermore, the level of phosphorylation of eNOS at Ser^1177^ and Thr^495^, relative to the total eNOS protein content, was comparable between wild‐type and *Gch1* ^*fl/fl*^Tie2cre mesenteric arteries (Figure 2C, E, F). Taken together, these findings demonstrate that endothelial cell *Gch1* deletion and subsequent BH4 deficiency markedly decrease vascular NO bioactivity in mesenteric arteries.

To determine whether this deficiency in endothelial cell BH4 leads to eNOS uncoupling in mesenteric arteries, we assessed endothelial generation of ROS (including superoxide and hydrogen peroxide), by DHE fluorescence in frozen sections of second‐order mesenteric arteries. We observed that under basal conditions, *Gch1* ^*fl/fl*^Tie2cre mesenteric arteries generated threefold more endothelium‐derived ROS production than wild‐type controls (Figure 2G, H). In the presence of L‐NAME, the level of endothelium‐derived ROS production was significantly reduced in *Gch1* ^*fl/fl*^Tie2cre mesenteric arteries when compared with untreated *Gch1* ^*fl/fl*^Tie2cre mesenteric arteries but was unchanged in wild‐type mesenteric arteries compared with untreated wild‐type mesenteric arteries (Figure 2G, H). We next determined whether endothelial cell BH4 deficiency led to compensatory changes in antioxidant proteins that may influence the vascular response in mesenteric arteries. Western blot analysis showed that the protein levels of antioxidant enzymes (Cu/ZnSOD, MnSOD, EcSOD and catalase) were comparable between *Gch1* ^*fl/fl*^Tie2cre and wild‐type controls (Figure S1A, B), indicating that deficiency in endothelial cell BH4 biosynthesis had no significant effect on antioxidant protein expression in mesenteric arteries. Taken together, these data demonstrate that endothelial cell BH4 deficiency leads to eNOS uncoupling, reduced NO bioactivity and increased ROS production in mesenteric arteries of *Gch1* ^*fl/fl*^Tie2cre mice.

### Endothelial cell‐specific BH4 deficiency leads to enhanced vasoconstriction and impaired vasodilatation in Gch1^fl/fl^Tie2cre mice

**Figure 3:**
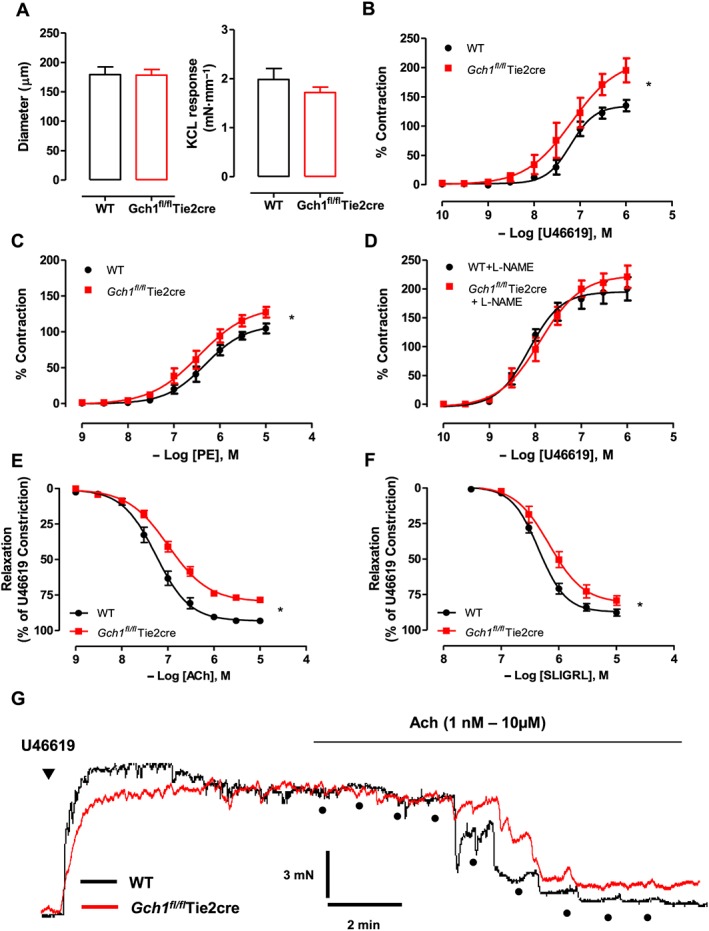
Vasomotor function in mesenteric arteries from *Gch1* ^*fl/fl*^Tie2cre and WT littermates. Isometric tension studies in isolated second‐order mesenteric arteries from WT and *Gch1* ^*fl/fl*^Tie2cre mice were determined using a wire myograph. (A) Diameter of mesenteric arteries and maximal contraction to 45 mmol·L^−1^ KCl. (B) Receptor‐mediated vasoconstriction to TxA_2_ mimetic, U46619 and (C) phenylephrine (PE). Data are presented as % of maximum KCL constriction to control for variation in vessel size. (D) Vasoconstriction to U46619 in the presence of NOS inhibitor, L‐NAME (100 μmol·L^−1^). (E) Receptor‐mediated endothelium‐dependent vasodilatation to ACh and (F) to the PAR2 agonist, SLIGRL. *P < 0.05; significantly different from WT; *n* = 8 WT and 10 *Gch1* ^*fl/fl*^Tie2cre, one WT mice was re‐genotyped as a *Gch1* ^*fl/fl*^Tie2cre mouse and re‐assigned at the end of the experiment). (G) Original traces demonstrating concentration‐dependent vasodilatation evoked by ACh.

We next investigated the requirement for endothelial cell BH4 biosynthesis on vasomotor function in mesenteric arteries. The diameters of the second order mesenteric arteries, as determined by length‐tension relationship, were comparable between wild‐type and *Gch1* ^*fl/fl*^Tie2cre mice (Figure 3A). No difference was detected between the contraction response to 45 mmol·L^−1^ KCl in wild‐type and *Gch1* ^*fl/fl*^Tie2cre arteries (Figure 3A). Receptor‐mediated vasoconstrictions to the TxA_2_ receptor agonist, U46619, were significantly enhanced in *Gch1* ^*fl/fl*^Tie2cre mesenteric arteries compared to wild‐type controls (Figure 3B). Receptor‐mediated vasoconstrictions to phenylephrine were also markedly enhanced in *Gch1* ^*fl/fl*^Tie2cre mesenteric arteries compared to wild‐type controls (Figure 3C), suggesting that the enhanced vasoconstriction was not due to specific alteration of receptor signalling in endothelial cells of *Gch1* ^*fl/fl*^Tie2cre mice. This difference was abolished in the presence of the NOS inhibitor, L‐NAME (Figure 3D), suggesting loss of basal eNOS‐derived NO in mesenteric arteries of *Gch1* ^*fl/fl*^Tie2cre mice.

Receptor‐mediated endothelium‐dependent vasodilatations to ACh (Figure 3E, G) or the PAR2 agonist, SLIGRL, (Figure 3F) were both significantly impaired in mesenteric arteries from *Gch1* ^*fl/fl*^Tie2cre mice, suggesting that the impaired vasodilatations were not due to specific alteration of receptor signalling in endothelial cells of *Gch1* ^*fl/fl*^Tie2cre mice. Endothelium‐independent vasodilatations to the NO donor, SNP were comparable between genotypes (Figure S2A), indicating the impairment of vasodilation in *Gch1* ^*fl/fl*^Tie2cre mesenteric arteries was not due to the alteration of vascular smooth muscle sensitivity. Taken together, our results showed that deficiency in endothelial cell BH4 biosynthesis leads to a loss of eNOS‐derived NO‐mediated vascular tone and impaired endothelium‐dependent vasodilatation in *Gch1* ^*fl/fl*^Tie2cre mesenteric arteries.

### Contribution of NO, prostacyclin, and EDHF in mesenteric arteries from mice deficient in endothelial cell BH4

**Figure 4:**
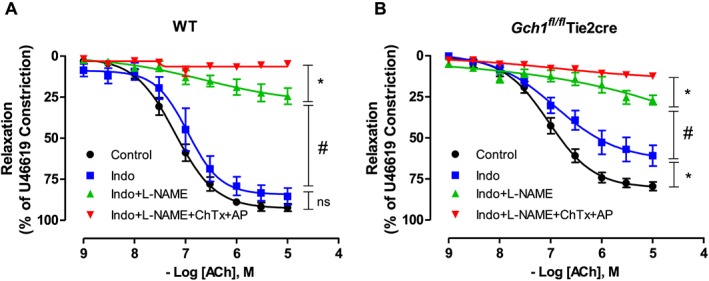
Contribution of cyclooxygenase‐derived vasodilators, eNOS‐derived vasodilators and EDHF in mouse mesenteric arteries. Isometric tension of second‐order mesenteric arteries from (A) WT and (B) *Gch1* ^*fl/fl*^Tie2cre mesenteric arteries was recorded**.** Endothelium‐dependent vasodilatations to ACh were determined in the presence of the cyclooxygenase inhibitor, indomethacin (10 μmol·L^−1^) alone or indomethacin with the NOS inhibitor, L‐NAME (100 μmol·L^−1^) or indomethacin, L‐NAME with EDHF blockers (apamin; AP and charybdotoxin; ChTx). Data are expressed as mean ± SE; *n* = 8 WT and 10 *Gch1* ^*fl/fl*^Tie2cre, one WT mice was re‐genotyped as a Gch1fl/flTie2cre mouse and re‐assigned at the end of the experiment). *P < 0.05, #*P* < 0.05; significantly different as indicated.

We next determined the relative contributions of the endothelium‐derived vasodilators NO, prostacyclin and EDHF in mesenteric arteries of mice lacking endothelial cell BH4. The contribution of NO, prostacyclin, and EDHF were determined by the inhibitory effect of L‐NAME (100 μmol·L^−1^), indomethacin (10 μmol·L^−1^), and EDHF blockers: apamin (small‐conductance Ca^2+^‐activated K^+^ channels blocker; 50 nmol·L^−1^) and charybdotoxin (non‐selective intermediate and large‐conductance Ca^2+^‐activated K^+^ channels blocker; 100 nmol·L^−1^) respectively. In mesenteric arteries of wild‐type mice, endothelium‐dependent vasodilatations in response to ACh were unaltered by indomethacin alone but significantly inhibited by indomethacin and L‐NAME together, and totally abolished by the combination of indomethacin, L‐NAME, apamin and charybdotoxin (Figure 4A). These data suggest that eNOS‐derived vasodilators and non‐eNOS‐derived EDHF play an important role in modulating vascular function in mouse mesenteric arteries whereas cyclooxygenase‐derived products do not. In mesenteric arteries of *Gch1* ^*fl/fl*^Tie2cre mice, endothelium‐dependent vasodilatations in response to ACh were significantly inhibited by indomethacin, and markedly attenuated by indomethacin and L‐NAME, and totally abolished by indomethacin and L‐NAME combined with apamin and charybdotoxin (Figure 4B). Importantly, the relative contribution of eNOS‐derived vasodilators (observed in the presence of indomethacin) was markedly reduced in mesenteric arteries of *Gch1* ^*fl/fl*^Tie2cre mice compared to wild‐type control mice, further suggesting the loss of eNOS‐derived NO is responsible for the impairment in *Gch1* ^*fl/fl*^Tie2cre mesenteric arteries.

### Increased endothelial eNOS‐derived H_2_O_2_‐mediated vasodilatation in mesenteric arteries from Gch1^fl/fl^Tie2cre mice

**Figure 5:**
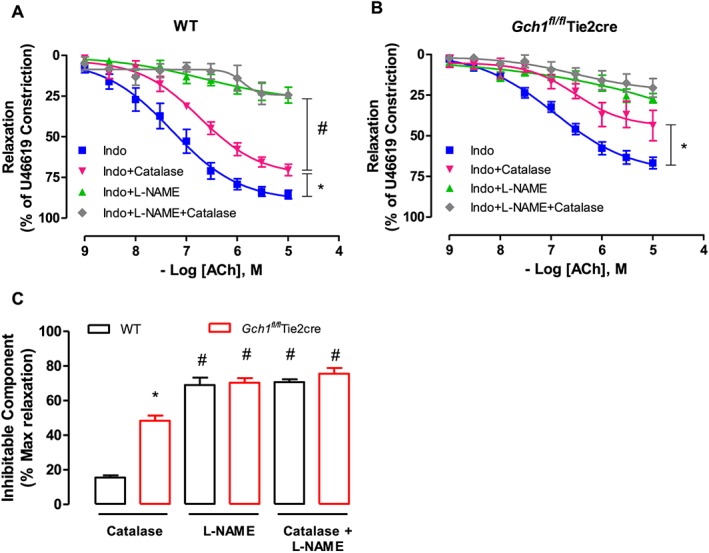
Contribution of eNOS‐derived H_2_O_2_ in mouse mesenteric arteries. Isometric tension of second‐order mesenteric arteries from (A) WT and (B) *Gch1* ^*fl/fl*^Tie2cre mesenteric arteries was recorded. Endothelium‐dependent vasodilatation to ACh was determined in the presence of the cyclooxygenase inhibitor, indomethacin (10 μmol·L^−1^) alone, indomethacin with PEG‐catalase (400 units·mL^−1^), indomethacin with the NOS inhibitor, L‐NAME (100 μmol·L^−1^), indomethacin and L‐NAME with PEG‐catalase. Data are expressed as mean ± SEM (*n* = 8 to 10 animals per group). *P < 0.05, #*P* < 0.05; significantly different as indicated. (C) Inhibitable‐component, expressed as percentage maximum relaxation in the presence of indomethacin, after treatment of PEG‐catalase, or L‐NAME, or PEG‐catalase with L‐NAME. *P < 0.05, significantly different from WT; #*P* < 0.05, significantly different from catalase treatment of the same genotype; *n* = 8 WT and 10 *Gch1* ^*fl/fl*^Tie2cre, one WT mice was re‐genotyped as a *Gch1* ^*fl/fl*^Tie2cre mouse and re‐assigned at the end of the experiment).

We next investigated whether endothelium‐dependent vasodilatation in mouse mesenteric arteries is in part mediated by eNOS‐derived H_2_O_2_ (Matoba *et al*., 2000; Prysyazhna *et al*., 2012). Consistent with previous reports, PEG‐catalase (400 units·mL^−1^), an enzyme that dismutates H_2_O_2_ to form water and oxygen, significantly inhibited endothelium‐dependent vasodilatations to ACh in mesenteric arteries of wild‐type (~20% inhibition of total maximum relaxation) and *Gch1* ^*fl/fl*^Tie2cre mice (~50% inhibition of total maximum relaxation) in the presence of indomethacin (Figure 5A–C). This concentration of PEG‐catalase (400 units·mL^−1^) was sufficient to inhibit vascular H_2_O_2_ production, as shown by the fact that additional application of PEG‐catalase (1000 units·mL^−1^) had no further inhibitory effect on the PEG‐catalase resistant vasodilatation in either wild‐type or *Gch1* ^*fl/fl*^Tie2cre mesenteric arteries (*n* = 4, data not shown). In the presence of indomethacin and L‐NAME, ACh‐induced vasodilatation was almost completely abolished in both wild‐type and *Gch1* ^*fl/fl*^Tie2cre vessels, and the addition of PEG‐catalase had no further inhibitory effect on ACh‐induced vasodilatation (Figure 5A–C), indicating that eNOS is the principal source of H_2_O_2_ in mesenteric arteries of both wild‐type and *Gch1* ^*fl/fl*^Tie2cre mice. In addition, endothelium‐independent vasodilatations to exogenous H_2_O_2_ were comparable between wild‐type and *Gch1* ^*fl/fl*^Tie2cre mesenteric arteries (Figure S2B). Taken together, these results demonstrate that uncoupled eNOS increases vascular H_2_O_2_ in mesenteric arteries of mice, deficient in endothelial cell BH4.

### Effect of superoxide dismutase and sepiapterin on endothelium‐dependent vasodilatation of mesenteric arteries from in Gch1^fl/fl^Tie2cre and wild‐type mice

**Figure 6:**
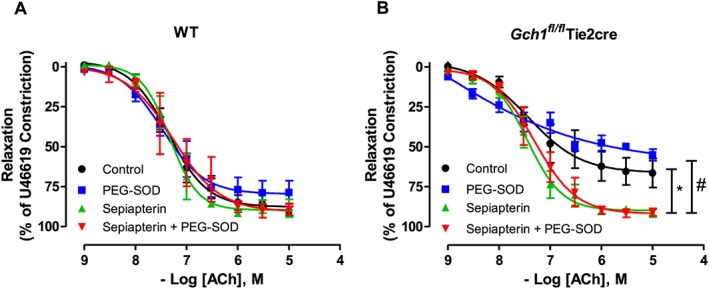
Effect of superoxide dismutase and sepiapterin on endothelium‐dependent vasodilatation in *Gch1* ^*fl/fl*^Tie2cre mesenteric arteries. Endothelium‐dependent vasodilatation in response to ACh in the presence of PEG‐SOD (200 units·mL^−1^) alone, or sepiapterin (100 μmol·L^−1^) alone or sepiapterin with PEG‐SOD in (A) WT and (B) *Gch1* ^*fl/fl*^Tie2cre mesenteric arteries. Data are expressed as mean ± SEM (*n* = 5 WT and six *Gch1* ^*fl/fl*^Tie2cre, one vessel from a WT mouse was not viable). *P < 0.05, significant difference between control and sepiapterin; #*P* < 0.05, significant difference between PEG‐SOD and sepiapterin + PEG‐SOD.

We next determined whether reducing vascular superoxide generation with the superoxide scavenger PEG‐SOD, thereby increasing tonic NO bioavailability, would be sufficient to restore endothelium‐dependent vasodilatation in *Gch1* ^*fl/fl*^Tie2cre mesenteric arteries. Pre‐incubation of PEG‐SOD (200 units·mL^−1^) had no significant effect on either sensitivity or maximal vasodilatation to ACh in mesenteric arteries from either *Gch1* ^*fl/fl*^Tie2cre mice or wild‐type mice (Figure 6A, B). This finding suggests that a decreased vascular superoxide generation alone is not sufficient to rescue vascular dysfunction in mesenteric arteries from *Gch1* ^*fl/fl*^Tie2cre mice.

We next sought to determine if we could rescue endothelial function in mesenteric arteries from *Gch1* ^*fl/fl*^Tie2cre with exogenous supplementation with sepiapterin, which augments BH4 levels via the salvage pathway independently of *de novo* BH4 biosynthesis by GTPCH. Pre‐incubation with sepiapterin (100 μmol·L^−1^) significantly enhanced the sensitivity and maximal vasodilatation to ACh in mesenteric arteries from *Gch1* ^*fl/fl*^Tie2cre mice (Figure 6A, B) but had no significant effect on vasodilatation of mesenteric arteries from wild‐type mice (Figure 6A, B). Pre‐incubation with sepiapterin in combination with PEG‐SOD did not further improve endothelium‐dependent vasodilatations in either *Gch1* ^*fl/fl*^Tie2cre or wild‐type mice. This finding suggests that reduced vascular superoxide generation alone, without increasing vascular BH4 bioavailability, is not sufficient to restore endothelium‐dependent vasodilatation in *Gch1* ^*fl/fl*^Tie2cre mice.

### Effect of endothelial cell BH4 deficiency on the ratio of eNOS dimers to monomers and on eNOS‐glutathionylation

**Figure 7:**
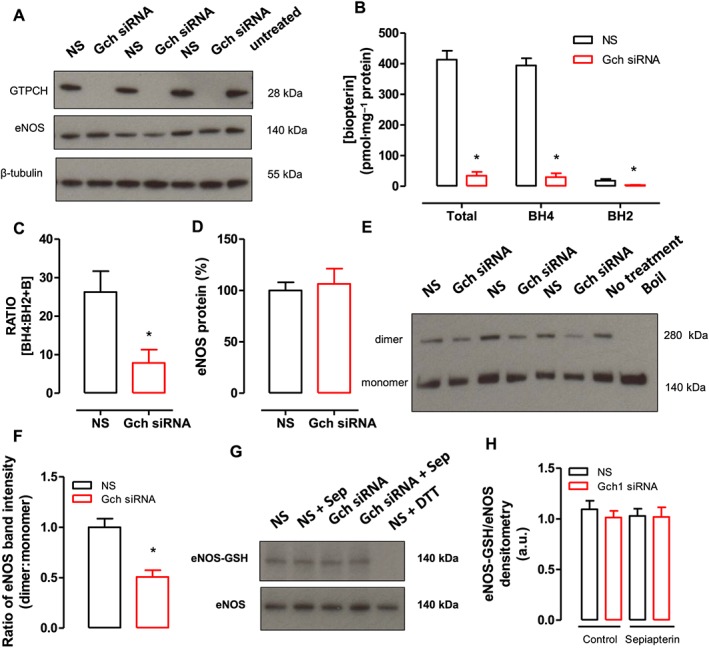
Effect of endothelial cell BH4 deficiency on the ratio of eNOS dimers to monomers and eNOS glutathionylation. (A) Representative Western blot for GTPCH, eNOS protein in sEND.1 mouse endothelial cell line treated with non‐specific *Gch1* siRNA (NS) or specific *Gch1* siRNA (*Gch1* siRNA). (B) Intracellular BH4, BH2 and total biopterins, measured by HPLC, were significantly reduced in *Gch1* specific siRNA cells compared with non‐specific siRNA cells. (C) Ratio of BH4 relative to oxidized biopterin species (BH4:BH2 + B). (D) Quantification data, measured as band density of eNOS to β‐tubulin. (E) Representative Western blot for eNOS dimer/monomer in sEND.1 mouse endothelial cell line treated with non‐specific *Gch1* siRNA (NS) or specific *Gch1* siRNA (*Gch1* siRNA) using a low‐temperature gel. (F) Quantification data, measured as band density of dimer to monomer protein in NS and *Gch1* siRNA. Western blot analyses are representative of three separate experiments. (G, H) Representative Western blot for eNOS‐glutathionylation and eNOS in sEND.1 with non‐specific and specific siRNA knockdown with or without supplementation of 10 μmol·L^−1^ sepaipterin by immunoprecipitation. Non‐specific sEND.1 knockdown with dithiothreitol (DTT; 1 mmol·L^−1^) was used as negative control for eNOS‐glutathionylation. Western blot analyses are representative of six separate experiments. Data are expressed as mean ± SEM; *n* = 6. *P < 0.05, significantly different from non‐specific siRNA).

We next determined the effect of endothelial cell BH4 deficiency on eNOS homodimer:monomer ratio using the mouse endothelial cell line (sEND.1) with *Gch1*‐specific siRNA knockdown. We found that transfection of sEND.1 cells with *Gch1*‐specific siRNA leads to a substantial decrease in GTPCH protein expression detected by Western blotting (Figure 7A). Correspondingly, intracellular BH4 levels were significantly decreased by ~90% in *Gch1‐*specific siRNA cells compared with non‐specific siRNA control cells (Figure 7B). The ratio of BH4 relative to oxidized biopterin species (BH4:BH2 + B) was significantly reduced in *Gch1‐*specific siRNA cells compared with non‐specific siRNA control cells (Figure 7C). In addition, there was no significant difference in eNOS protein expression between the groups (Figure 7D). Following low‐temperature Western blotting, we found that the eNOS dimer:monomer ratio was significantly decreased in *Gch1‐*specific siRNA cells compared with non‐specific siRNA cells, indicating uncoupling of eNOS. This finding demonstrates that endogenous BH4 plays a key role in promoting and stabilizing eNOS protein monomers into the active homodimeric form of the enzyme (Figure 7E, F).

We then next investigated whether eNOS‐glutathionylation is affected by eNOS uncoupling in endothelial cell BH4 deficiency. Using *Gch1*‐specific siRNA knockdown in sEND.1 cells, we observed that there was no significant difference in eNOS‐glutathionylation, relative to the total eNOS protein content, between non‐specific siRNA cells and specific *Gch1* siRNA cells (Figure 7G, H). Supplementation with the BH4 analogue, sepiapterin (10 μM, 24 h) did not alter eNOS‐glutathionylation status, in either non‐specific siRNA cells or specific *Gch1* siRNA cells. Taken together, these data demonstrate that eNOS‐glutathionylation is unlikely to be influenced by intracellular BH4 levels in mouse endothelial cells in this experimental setting (Figure 7G, H), and perhaps eNOS uncoupling predominately occurs at the reductase domain in endothelial cells, as shown by the ability of L‐NAME to prevent superoxide production from eNOS.

## Discussion

In this study, we have investigated the physiological requirement for endothelial cell BH4‐dependent eNOS regulation in the vascular responses of resistance arteries. The major findings of this study are as follows. First, endothelium‐targeted *Gch1* deletion leads to endothelial cell BH4 deficiency, eNOS uncoupling, increased ROS production and loss of eNOS‐derived NO‐mediated vascular tone, resulting in vascular dysfunction. Second, endothelial cell BH4 deficiency leads to an increase in eNOS‐derived H_2_O_2_ generation that mediates endothelium‐dependent vasodilatation in mesenteric arteries from *Gch1* ^*fl/fl*^Tie2cre mice. Third, loss of eNOS‐derived NO‐mediated vasodilatation is associated with up‐regulation of cyclooxygenase‐derived vasodilators. Taken together, these studies demonstrate that endothelial cell BH4‐dependent eNOS regulation plays a pivotal role in maintaining vasomotor homeostasis in resistance arteries and identifies endothelial cell BH4 as a key regulator of eNOS‐derived NO versus eNOS‐derived H_2_O_2_ as alternative endothelium‐derived relaxing factors.

In this study, we have demonstrated that deficiency in endothelial cell BH4 biosynthesis in mesenteric arteries leads to eNOS uncoupling, a loss of eNOS‐derived NO, enhanced vasoconstriction and impaired endothelium‐dependent vasodilatation in *Gch1* ^*fl/fl*^Tie2cre mice. This observation is consistent with previous reports, which have reported that reduced vascular BH4 bioavailability leads to eNOS uncoupling and loss of NO‐mediated vasodilatations in mesenteric arteries of DOCA‐salt hypertension mice (Du *et al*., 2008). This effect was prevented in transgenic mice with endothelial‐specific GTPCH overexpression (GCH‐Tg)‐DOCA‐salt hypertension mice (Du *et al*., 2008). Although these studies implicate reduced or augmented BH4 in vascular disease pathogenesis, the physiological requirement for endothelial cell BH4 biosynthesis in the regulation of eNOS‐derived vasodilators in vasomotor function in resistance arteries remained unknown. In the present study, we have demonstrated that deficiency in endothelial cell BH4 biosynthesis is alone sufficient to cause vascular dysfunction in resistance arteries, even in the absence of vascular disease, suggesting the important role of endothelial cell BH4‐dependent eNOS regulation in modulating vascular tone in mesenteric arteries.

It is unlikely that down‐regulation of eNOS protein expression is responsible for reduced NO‐mediated vasodilatation because eNOS expression was unaltered in *Gch1* ^*fl/fl*^Tie2cre mesenteric arteries. In addition, this difference did not appear to be due to alteration in the phosphorylation status of eNOS or the induction of eNOS‐glutathionylation, although parallel experiments in endothelial cells may suggest that the presence of BH4 stabilizes the eNOS dimer.

Despite impairment of vascular function in *Gch1* ^*fl/fl*^Tie2cre mesenteric arteries, vessel diameter and contractile response to KCl were comparable between the genotypes suggesting that the enhanced contractile response was not due to structural differences in the arteries between groups. Endothelium‐independent vasodilatations in response to SNP and exogenous H_2_O_2_ were also comparable, indicating that the vasodilatation properties of vascular smooth muscle cells were preserved in *Gch1* ^*fl/fl*^Tie2cre mice and wild‐type littermate controls, and this impairment was specific to the endothelium.

We found that endothelium‐dependent vasodilatation was in part mediated by eNOS‐derived H_2_O_2_ in both wild‐type and *Gch1* ^*fl/fl*^Tie2cre mesenteric arteries. This finding is consistent with previous reports in mouse and human mesenteric arteries, as well as porcine coronary arteries (Matoba *et al*., 2000; Matoba *et al*., 2002; Prysyazhna *et al*., 2012). eNOS‐deficient mice have markedly reduced eNOS‐derived H_2_O_2_‐mediated vasodilation in mesenteric arteries (Matoba *et al*., 2000; Takaki *et al*., 2008). Furthermore, mice with deficiency in Cu/ZnSOD lack eNOS‐derived H_2_O_2_ generation and exhibit impaired ACh‐induced vasodilation in mesenteric arteries, further suggesting a key role of eNOS‐derived H_2_O_2_‐mediated vasodilation in mesenteric arteries. Interestingly, we have demonstrated that almost 50% of the remaining vasodilatation in *Gch1* ^*fl/fl*^Tie2cre mesenteric arteries was mediated by eNOS‐derived H_2_O_2_, compared with ~20% in wild‐type mesenteric arteries. Consistent with these results, endothelial ROS production assessed by DHE fluorescence in mesenteric arteries was significantly increased in *Gch1* ^*fl/fl*^Tie2cre mice, which was inhibited by L‐NAME, suggesting that eNOS is a principal source of ROS production in *Gch1* ^*fl/fl*^Tie2cre mesenteric arteries. This finding indicates that deficiency in endothelial cell BH4 biosynthesis leads to eNOS uncoupling and increased eNOS‐derived H_2_O_2_‐mediated vasodilation in resistance arteries. However, critically, this compensatory increase in H_2_O_2_‐mediated vasodilation was not sufficient to rescue the endothelial cell dysfunction observed in *Gch1* ^*fl/fl*^Tie2cre mice. Consistent with this finding, we have recently reported that endothelium‐dependent vasodilatation was at least in part mediated by eNOS‐derived H_2_O_2_ in aortas from endothelial cell‐specific BH4 deficient mice (Chuaiphichai *et al*., 2014). It is unlikely that alteration in the antioxidant protein abundance contributed to this observation because the levels of antioxidant proteins (MnSOD, Cu/ZnSOD, ecSOD and catalase) were all comparable between *Gch1* ^*fl/fl*^Tie2cre and wild‐type mesenteric arteries. This finding demonstrates for the first time that endothelial cell BH4 bioavailability is the key determinant of eNOS‐derived NO versus eNOS‐derived H_2_O_2_ in resistance arteries.

Interestingly, approximately 20% of the L‐NAME‐inhibitable component of relaxation was still observed in the presence of indomethacin and catalase in *Gch1* ^*fl/fl*^Tie2cre mesenteric arteries, suggesting that another eNOS‐derived vasodilator (e.g. nitroxyl anion) is mediating vasodilatation in these vessels. Several reports demonstrate that eNOS uncoupling may also be the source of nitroxyl anion, which may act as an additional eNOS‐derived compensatory dilator in vascular disease states (Kojda *et al*., 1994; Kojda and Harrison, 1999). Therefore, it is possible that nitroxyl anion from eNOS uncoupling may play a role in eNOS‐mediated vasodilatation in this study. Further studies are warranted to determine the contribution of eNOS‐derived nitroxyl anion in this model.

Endothelium‐dependent vasodilatation in *Gch1* ^*fl/fl*^Tie2cre mesenteric arteries was in part mediated by a cyclooxygenase‐derived vasodilator, probably prostacyclin. However, despite up‐regulation of a cyclooxygenase ‐derived vasodilator, endothelium‐dependent vasodilatation in *Gch1* ^*fl/fl*^Tie2cre mesenteric arteries was still markedly impaired compared with wild‐type controls. This finding suggests that when eNOS‐derived NO generation is compromised, cyclooxygenase‐derived prostacylin is up‐regulated to compensate in *Gch1* ^*fl/fl*^Tie2cre mesenteric arteries. This observation is consistent with previous reports, which have demonstrated that prostacyclin is up‐regulated in mesenteric arteries of eNOS knockout mice (Chataigneau *et al*., 1999; Matoba *et al*., 2000; Scotland *et al*., 2005). It has been suggested that endogenous eNOS‐derived NO acts as an inhibitor of prostacyclin synthesis. Indeed, Barker *et al.* have demonstrated that treatment of isolated rings of human saphenous vein with a NOS inhibitor increases the release of endogenous prostacyclin, which could be decreased by co‐administration with nitrogylcerin (Barker *et al*., 1996). These findings suggest that endothelial cell BH4/eNOS‐derived NO bioavailability plays a role in regulating the synthesis of endogenous prostacyclin in vascular physiology. Although cyclooxygenase‐derived vasodilators play some compensatory roles, as demonstrated in isometric tension studies of mesenteric arteries, the extent of the compensation was not enough to prevent basal blood pressure elevation in *Gch1* ^*fl/fl*^Tie2cre mice (Chuaiphichai *et al*., 2014).

In addition, we also observed that the remaining component of endothelial‐dependent dilation, which was not inhibited by indomethacin or by the combination of indomethacin with L‐NAME or by indomethacin combined with L‐NAME and catalase, was totally abolished in the presence of additional EDHF blockers: apamin and charybdotoxin. This suggests that there is a sizable proportion of the vasodilatation in mesenteric arteries of both genotypes, that is mediated by an EDHF that is not derived from eNOS or from cyclooxygenase. Currently, the possible candidates for this EDHF include C‐type natriuretic peptide (Chauhan *et al*., 2003), K^+^ ions (Edwards *et al*., 1998), epoxyeicosatrienoic acids (Campbell *et al*., 2001; Archer *et al*., 2003), hydrogen sulphide (H_2_S)(Mustafa *et al*., 2011) and myoendothelial gap junctions (Dora *et al*., 2003). The nature of remaining EDHF in this study is unlikely to be affected by the bioavailability of endothelial cell BH4 because the relative contribution of this remaining EDHF‐mediated vasodilatation was similar between wild‐type and *Gch1* ^*fl/fl*^Tie2cre mesenteric arteries.

To test the specificity of the observed phenotype for biochemical BH4 deficiency, we performed a rescue experiment using a BH4 precursor, sepiapterin. We have demonstrated that *ex vivo* supplementation of mesenteric arteries with sepiapterin restored endothelium‐dependent vasodilatation in *Gch1* ^*fl/fl*^Tie2cre mice. This result is consistent with previous reports, demonstrating that supplement with sepiapterin in hypertensive mice (Landmesser *et al*., 2003) and patients with diabetes (Guzik *et al*., 2002) improved NO bioavailability and restored endothelial function. Furthermore, reduced vascular eNOS‐derived ROS generation by PEG‐SOD had no additional effect on endothelium‐dependent vasodilatation in mesenteric arteries from *Gch1* ^*fl/fl*^Tie2cre mice in the presence or absence of sepiapterin, suggesting direct effect of BH4 on coupling eNOS rather than a non‐specific antioxidant effect of sepiapterin. This finding suggests, for the first time, that reducing vascular ROS generation alone without increasing endothelial cell BH4 bioavailability is not sufficient to restore endothelial function in resistance arteries from *Gch1* ^*fl/fl*^Tie2cre mice. This may be one of the reasons why non‐selective antioxidant drugs in large‐scale clinical trials have failed to show any health benefit for the treatment of cardiovascular disease. Therefore, antioxidants may need to be combined with BH4 supplementation to provide benefit in the treatment of vascular disease.

In summary, we have demonstrated for the first time that selective deficiency in endothelial cell BH4 biosynthesis, by targeted *Gch1* deletion, is alone sufficient to cause eNOS uncoupling, loss of eNOS‐derived NO, increased eNOS‐derived H_2_O_2_ and impaired vascular function in resistance arteries even in the absence of vascular disease. These findings suggest that endothelial cell BH4‐dependent eNOS regulation plays a pivotal role in maintaining vascular homeostasis in resistance arteries. Thus, targeting endothelial cell *Gch1* and BH4 biosynthesis may provide a therapeutic target for the treatment of vascular diseases.

## Author contributions

S.C., G.D. and K.M.C. designed the experiments. S.C., M.J.C., L.T., A.B.H. and G.D. performed the experiments, analysed and interpreted the data. E.M. and M.J.C. provided discussions. S.C., G.D. and K.M.C. wrote the manuscript. All authors contributed to editorial changes in the manuscript.

## Conflict of interest

The authors declare no conflicts of interest.

## Declaration of transparency and scientific rigour

This Declaration acknowledges that this paper adheres to the principles for transparent reporting and scientific rigour of preclinical research recommended by funding agencies, publishers and other organisations engaged with supporting research.

## Supporting information


**Figure S1** (A) Representative immunoblots showing catalase, EcSOD, Cu/ZnSOD and MnSOD protein in wild‐type and *Gch1* ^*fl/fl*^Tie2cre mesenteric arteries, (B) with quantitative data, measured as percentage band density and corresponding β‐tubulin (*n* = 6 animals per group).

**Figure S2** (A) Endothelium‐independent vasodilatation to sodium nitroprusside (SNP; *n* = 7 to 9 animals per group) and (B) hydrogen peroxide (H_2_O_2_; *n* = 4 to 5 animals per group). Data are expressed as mean ± SE.
